# Perforating Veins Detected by Endoscopic Ultrasonography Are Useful in Predicting the Recurrence of Esophageal Varices After Endoscopic Variceal Ligation Combined With Argon Plasma Coagulation

**DOI:** 10.1111/den.70132

**Published:** 2026-03-09

**Authors:** Yukari Tezuka, Jun Takada, Takao Miwa, Kiichi Otani, Naoya Masuda, Hiroki Taniguchi, Kentaro Kojima, Sachiyo Onishi, Masaya Kubota, Takashi Ibuka, Masahito Shimizu

**Affiliations:** ^1^ Department of Gastroenterology/Internal Medicine, Graduate School of Medicine Gifu University Gifu Japan; ^2^ Department of Medicine University of California San Diego La Jolla California USA

**Keywords:** after treatment, endoscopic ultrasonography, portal hypertension, safety management, secondary prevention

## Abstract

**Objectives:**

Few attempts have been made to determine the risk factors for the recurrence of esophageal varices (EV) and the optimal surveillance interval. This study analyzed whether endoscopic ultrasonography can be used to predict EV recurrence and determine the optimal timing for surveillance endoscopy post‐treatment.

**Methods:**

We retrospectively evaluated patients with EVs who underwent endoscopic variceal ligation (EVL) combined with argon plasma coagulation (APC), followed by endoscopic ultrasonography (EUS) using a miniature ultrasonic probe 1 month after APC. Factors associated with EV recurrence were assessed using the Fine‐Gray competing risk regression model, with death considered as the competing risk. The cumulative incidence of EV recurrence was estimated using the cumulative incidence function, and groups were compared using Gray's test.

**Results:**

Of 163 eligible patients, 37 (23%) experienced EV recurrence during a median follow‐up period of 36 months (interquartile range: 15–76 months). Multivariable analysis revealed that the presence of perforating veins (PVs) was a significant factor for EV recurrence (sub‐distribution hazard ratio, 4.30; 95% confidence interval, 2.13–8.71; *p* < 0.001). The cumulative incidence of EV recurrence was significantly higher in patients with PV than in those without (6‐month and 1‐year recurrence rates: 27.3% and 41.6% vs. 3.9% and 5.5%, respectively; *p* < 0.001).

**Conclusions:**

PVs detected using EUS independently predict EV recurrence risk after EVL combined with APC. Given that patients with PVs experience a high recurrence rate within 6 months, comprehensive surveillance endoscopy at 6‐month intervals is recommended in the first year post‐treatment.

## 1 Introduction

The recurrence of esophageal varices (EVs) after endoscopic treatment remains a significant clinical issue [1, 2]. Notably, EV recurrence is accompanied by variceal bleeding, leading to a high mortality rate in these patients [3, 4]; therefore, its detection and treatment before fatal clinical events occur are necessary. Endoscopic variceal ligation (EVL) is a globally used method for treating EVs; moreover, additional consolidation therapy using argon plasma coagulation (APC) has been reported to decrease recurrence compared with EVL alone [2, 5, 6]. Although EVL combined with APC is an effective treatment, the recurrence risk remains high, requiring the urgent identification of risk factors for recurrence to conduct effective endoscopic surveillance of high‐risk patients after this treatment.

The American Society for Gastrointestinal Endoscopy (ASGE) and the European Society of Gastrointestinal Endoscopy (ESGE) recommend surveillance esophagogastroduodenoscopy (EGD) after EV eradication [7, 8]. However, evidence supporting this interval remains unclear; importantly, these guidelines do not provide a detailed rationale for it. Endoscopic ultrasonography (EUS) is an effective method for identifying patients at risk of post‐treatment EV recurrence. Moreover, a prior study reported that peri‐esophageal veins (peri‐V) ≥ 2 mm and large perforating veins (PVs) of the esophagus, detected by EUS, in patients undergoing endoscopic injection sclerotherapy, are significant factors for EV recurrence [9]. Therefore, we hypothesized that EUS findings could predict EV recurrence after EVL combined with APC. Additionally, determining the duration of recurrence in high‐risk populations can help determine the optimal interval for performing endoscopic surveillance in these patients.

This study aimed to clarify the usefulness of EUS findings in predicting EV recurrence in patients after EVL combined with APC. Furthermore, the time to recurrence was investigated to derive the optimal timing for surveillance endoscopy after EV treatment.

## 2 Methods

### 2.1 Study Design and Patients

This retrospective cohort study reviewed patients with EV who underwent EVL combined with APC for ruptured or high‐risk EV at Gifu University Hospital between January 2013 and October 2023. The inclusion criteria were patients aged ≥ 18 years with EV treated with EVL combined with APC, who underwent EUS after these treatments. Both patients receiving treatment for primary and secondary prophylaxis were included. The exclusion criteria included patients who did not undergo APC, EUS, or both, and those with unclear EUS findings.

### 2.2 Details of EVL Combined With APC

A GIF‐H290 or GIF‐Q260J electronic endoscope (Olympus Co., Tokyo, Japan) was used for EVL, APC, and EUS. A pneumoactivated EVL device (SB‐KAWASUMI LABORATORIES Inc., Kanagawa, Japan) was employed. EVs were ligated from the esophagogastric junction (EGJ) to the mid‐esophagus, and sessions were repeated every 2–4 weeks until EVs were eradicated. Following endoscopic confirmation of eradication, defined as F0 status without any submucosal elevations suggestive of varices, APC was performed just once about a month after the last EVL with an ERBE APC2 generator equipped with VIO3 or VIO300 units and FiAPC probes (ERBE Electromedizin, Tuebingen, Germany) (Figure S1). APC was delivered using intermittent energy mode (pulsed APC) with an argon flow of 2.0 L min^−1^ and a high‐frequency arc output of 20 W. Coagulation was applied from the EGJ to 5 cm orally. After circumferential mucosal cauterization, the coagulated tissue was scraped using an endoscope equipped with a tip attachment (Elastic Touch, Top, Tokyo, Japan). Additional coagulation was then performed on the remaining non‐coagulated areas to ensure complete coagulation across the entire region. This protocol was applied to all patients, none of whom underwent endoscopic sclerotherapy.

### 2.3 Details of the EUS Procedure and Follow‐Up

EUS and follow‐up endoscopy were performed 1 month after APC. EUS with a miniature ultrasonic probe (UM‐3R: 20 MHz; Olympus Co., Tokyo, Japan) was conducted over a 10‐cm range from the EGJ to the middle section of the esophagus with the lumen filled with water to search for collateral vessels inside and outside the esophageal wall; peri‐Vs were small vessels adjacent to the outside of the muscularis propria of the esophagus, and para‐esophageal veins (para‐Vs) were large vessels separated from the muscularis propria. The PVs penetrated the esophageal wall and connected with either the peri‐Vs or para‐Vs (Figure [Link], [Link]). Importantly, a high‐frequency 20‐MHz ultrasound miniprobe provides superior axial resolution for shallow structures. All EUS procedures were conducted by three expert endoscopists, each with > 10 years of experience and > 50 prior cases. Radial‐ and convex‐arrayed echoendoscopes were not used in the procedure. The findings were independently reviewed and interpreted by two board‐certified fellows of the Japan Gastroenterological Endoscopy Society. In patients with portal vein thrombosis, anticoagulant therapy was uniformly initiated after completion of all treatment protocols and subsequent EUS. Surveillance endoscopy was performed every 3–6 months by each endoscopist. When EV recurrence was confirmed, the EVL combined with APC treatment protocol was reapplied.

### 2.4 Outcome and Definitions

The primary outcome of this study was EV recurrence in patients who underwent EVL combined with APC. Endoscopic findings of EV were classified according to the General Rules for the Study of Portal Hypertension [10]. We defined high risk and recurrence as the development of more than F2 EVs or a positive red color sign.

### 2.5 Statistical Analyses

Data are presented as median (interquartile range [IQR]) for continuous variables and number (%) for categorical variables. The baseline characteristics of the groups were compared using the Mann–Whitney *U* test or Fisher's exact test. Given mortality as a competing risk, factors associated with EV recurrence were investigated using the Fine‐Gray competing risk regression model, and the outcomes were explained as sub‐distribution hazard ratios (SHRs) with 95% confidence intervals. Considering the multicollinearity and clinical relevance, age, sex, platelet count reflecting liver stiffness, and the Child–Pugh score—which has been reported to be associated with EV recurrence—were used as predetermined covariates in the multivariable analysis [11, 12]. The cumulative incidence curves of EV recurrence were estimated using the cumulative incidence function, and the groups were compared using Gray's test. Patients with missing data were excluded from the analysis; therefore, imputation was not performed. Statistical significance was set at *p* < 0.05. All statistical analyses were performed using EZR (Saitama Medical Center, Jichi Medical University, Saitama, Japan), which is a modified version of R commander designed to add statistical functions frequently used in biostatistics [13].

### 2.6 Ethics

The study protocol was reviewed and approved by the Institutional Review Board of the Graduate School of Medicine, Gifu University (approval number: 2023‐242; approval date: January 17, 2024). The study was conducted in accordance with the principles of the Declaration of Helsinki. Given the retrospective nature of the study, informed consent was obtained from all the participants using an opt‐out method.

## 3 Results

### 3.1 Baseline Characteristics

Of the 275 patients reviewed, 163 were included in the analysis (Figure S3). During a median follow‐up period of 36 months (IQR: 15–76 months), EV recurrence and death without recurrence were observed in 37 (23%) and 30 (18%) patients, respectively. Table 1 presents the baseline clinical and procedural characteristics of the included patients divided into recurrence and non‐recurrence groups. The median age of the patients was 67 years (IQR: 57–75 years), and 107 patients (66%) were male. Alcohol‐associated liver disease, viral infections, and “other” were the primary cirrhosis etiologies in 56 (34%), 43 (26%), and 64 (39%) patients, respectively. Hepatocellular carcinoma and portal vein thrombosis were present in 39 (24%) and 16 (10%) patients, respectively. Overall, 147 (90%) and 16 (10%) patients were treated with EV for prophylaxis and for rupture, respectively. Lm 103 (63%), F2 114 (70%), RC1 92 (56%), and Cb 150 (92%) accounted for the majority of varices before EVL. The median total number and total number of EVL sessions were 17 (IQR: 14–24) and two (IQR: 2–2), respectively. Thirty‐eight cases (23%) exhibited a history of EV treatment, meaning they were recurrent cases. Detailed baseline characteristics by prior treatment history are displayed in Table S1.

**TABLE 1:** Baseline characteristics of patients who underwent variceal treatment, divided by recurrence status.

Characteristic	All patients (*n* = 163)	No recurrence (*n* = 126)	Recurrence (*n* = 37)	*p* ^a^
Age (years)	67 (57–75)	67.5 (59–76)	60.0 (50–71)	0.005
Male, *n* (%)	107 (66)	80 (64)	27 (73)	0.329
Body mass index (kg/m^2^)	23.0 (20.6–25.7)	23.0 (20.9–25.3)	23.0 (20.1–26.6)	0.820
Etiology of cirrhosis, *n* (%)				
Alcohol	56 (34)	42 (33)	14 (38)	0.512
Viral	43 (26)	36 (29)	7 (19)	
Others	64 (39)	48 (38)	16 (43)	
Hepatocellular carcinoma, *n* (%)	39 (24)	33 (26)	6 (16)	0.275
Portal vein thrombosis, *n* (%)	16 (10)	11 (9)	5 (14)	0.363
Portosystemic shunts other than esophageal collateral veins, *n* (%)	90 (55)	67 (53)	23 (62)	0.452
Paraumbilical veins	50 (31)	38 (30)	12 (32)	0.841
Splenorenal shunts	59 (36)	48 (38)	11 (30)	0.437
Gastrorenal shunt	14 (9)	8 (6)	6 (16)	0.091
Mesenteric vein shunt	6 (4)	5 (4)	1 (3)	1.000
Non‐selective beta‐blockers intake, *n* (%)	2 (1)	2 (2)	0 (0)	1.000
Ascites (moderate or severe), *n* (%)	47 (29)	36 (29)	11 (30)	1.000
Hepatic encephalopathy, *n* (%)	2 (1)	2 (2)	0 (0)	1.000
Child–Pugh class (A/B/C), *n*	102/53/8	76/45/5	26/8/3	0.169
Child–Pugh score	6 (5–7)	6 (5–7)	6 (5–7)	0.868
International normalized ratio	1.09 (1.03–1.19)	1.09 (1.02–1.19)	1.10 (1.07–1.21)	0.328
Platelet (10^9^/L)	87 (60–120)	92 (62–125)	66 (51–95)	0.011
Creatinine (mg/dL)	0.71 (0.59–0.85)	0.74 (0.61–0.89)	0.65 (0.56–0.84)	0.061
Albumin (g/dL)	3.60 (3.20–3.90)	3.55 (3.20–3.90)	3.60 (3.30–3.90)	0.694
Bilirubin (mg/dL)	1.30 (0.90–1.70)	1.30 (0.90–1.60)	1.40 (1.00–1.70)	0.268
History of EVs treatment, *n* (%)	38 (23)	22 (18)	16 (43)	0.002
Varices before treatment				
Location (Li/Lm/Ls), *n*	31/103/29	25/77/24	6/26/5	0.663
Form (F1/F2/F3), *n*	19/114/30	11/92/23	8/22/7	0.089
Red color sign (0/1/2/3), *n*	36/92/25/10	27/73/20/6	9/19/5/4	0.526
Color (Cb/Cw), *n*	150/13	118/8	32/5	0.173
Rupture, *n* (%)	16 (10)	12 (10)	4 (11)	0.761
Total number of EVL	17 (14–24)	17 (14–23)	18 (14–26)	0.490
Total session of EVL	2 (2–2)	2 (2–2)	2 (2–2)	0.842
EUS findings				
Para‐esophageal veins, *n* (%)	123 (76)	93 (74)	30 (81)	0.515
Peri‐esophageal veins, *n* (%)	107 (66)	78 (62)	29 (78)	0.077
Perforating vein, *n* (%)	22 (14)	10 (8)	12 (32)	< 0.001

*Note:* Values are presented as number (percentage) or median (interquartile range).

Abbreviations: EUS, endoscopic ultrasound; EVL, endoscopic variceal ligation.

^a^ Groups were compared using Fisher's exact test or the Mann–Whitney *U* test.

Regarding EUS findings, 123 (76%), 107 (66%), and 22 (14%) para‐V, peri‐V, and PV were identified on EUS 1 month post‐APC, respectively. There were significant differences between the recurrence and non‐recurrence groups in terms of age, presence of PV on EUS, and platelet counts.

### 3.2 Independent Factors for EV Recurrence

In univariable analyses, the presence of PV was associated with EV recurrence. Conversely, age, platelet count, creatinine level, and the F2 form of varices before treatment demonstrated a negative association (Table 2). Multivariable analysis including age, sex, Child–Pugh score, platelet, presence of peri‐V, and presence of PV demonstrated that PV (SHR, 3.97; 95% CI, 1.99–7.92; *p* < 0.001) and platelet count (SHR, 0.92; 95% CI, 0.85–0.99; *p* = 0.029) were independent factors for recurrence of varices (Table 3). When performing multivariable analysis with the same covariates, excluding cases with prior EV treatment, only PV (SHR, 4.65; 95% CI, 1.94–11.16; *p* < 0.001) was an independent factor (Table S2).

**TABLE 2:** Univariable analysis for factors predicting variceal recurrence.

Characteristic	SHR (95% CI)	*p* ^a^
Age (years)	0.98 (0.96–1.00)	0.027
Male	1.36 (0.67–2.79)	0.400
Body mass index (kg/m^2^)	1.02 (0.92–1.13)	0.710
Etiology of cirrhosis		
Viral^b^	1.00	
Alcohol	1.79 (0.77–4.18)	0.180
Others	1.73 (0.75–4.01)	0.200
Hepatocellular carcinoma	0.52 (0.22–1.27)	0.150
Portal vein thrombosis	1.57 (0.62–3.99)	0.340
Portosystemic shunts other than esophageal collateral veins	1.359 (0.70–2.63)	0.360
Ascites	1.05 (0.52–2.11)	0.900
Hepatic encephalopathy	0 (0–0)	0
Child–Pugh class (A/B/C)		
A^b^	1.00	
B	0.64 (0.29–1.41)	0.270
C	1.55 (0.54–4.42)	0.410
Child–Pugh score	0.99 (0.81–1.21)	0.930
International normalized ratio	1.48 (0.23–9.63)	0.680
Platelet (10^9^/L)	0.91 (0.85–0.98)	0.008
Creatinine (mg/dL)	0.25 (0.07–0.95)	0.042
Albumin (g/dL)	1.13 (0.71–1.80)	0.620
Bilirubin (mg/dL)	1.06 (0.84–1.32)	0.640
Varices before treatment		
Location		
Li^b^	1.00	
Lm	1.33 (0.55–3.20)	0.530
Ls	1.01 (0.33–3.10)	0.990
Form		
F1^b^	1.00	
F2	0.44 (0.21–0.93)	0.033
F3	0.63 (0.24–1.67)	0.350
Red color sign		
0^b^	1.00	
1	0.64 (0.29–1.44)	0.280
2	0.54 (0.19–1.50)	0.230
3	1.21 (0.41–3.59)	0.740
Color (Cb/Cw), *n*		
Cb^b^	1.00	
Cw	2.10 (0.83–5.31)	0.120
Rupture	1.21 (0.39–3.77)	0.740
Total number of EVL	1.02 (0.99–1.04)	0.290
Total session of EVL	1.11 (0.84–1.46)	0.450
EUS findings		
Para‐esophageal veins	1.52 (0.65–3.54)	0.330
Peri‐esophageal veins	2.10 (0.96–4.60)	0.063
Perforating vein	4.38 (2.15–8.93)	< 0.001

Abbreviations: CI, confidence interval; EUS, endoscopic ultrasound; EVL, endoscopic variceal ligation; SHR, sub‐distribution hazard ratio.

^a^ Analysis was performed using the fine‐gray competing risk regression model.

^b^ Reference group.

**TABLE 3:** Multivariable model for predicting varices recurrence.

Characteristic	SHR (95% CI)	*p* ^a^
Age (years)	0.98 (0.96–1.00)	0.210
Male	1.35 (0.64–2.85)	0.430
Child–Pugh score	1.00 (0.82–1.21)	0.960
Platelet (10^9^/L)	0.92 (0.85–0.99)	0.029
Peri‐esophageal veins	1.42 (0.67–3.00)	0.360
Perforating vein	3.97 (1.99–7.92)	< 0.001

Abbreviations: CI, confidence interval; SHR, sub‐distribution hazard ratio.

^a^ Analysis was performed using the fine‐gray competing risk regression model.

### 3.3 Cumulative Incidence and Duration of EV Recurrence in Patients With and Without PVs

**FIGURE 1:**
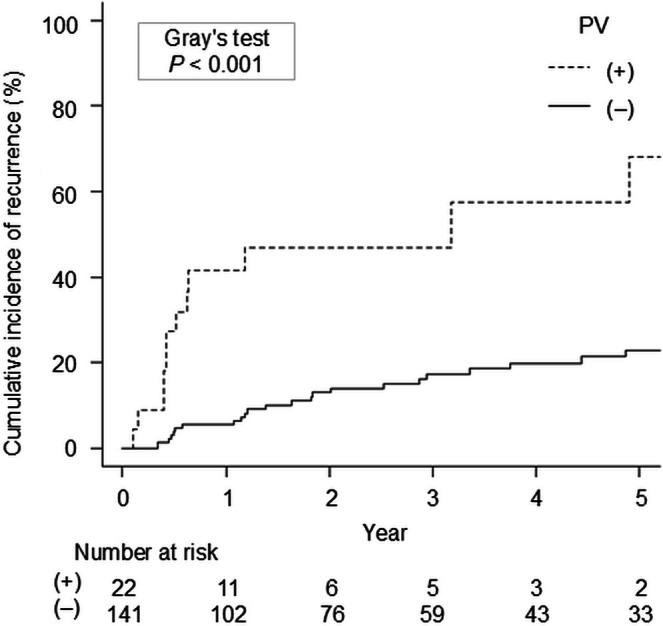
Cumulative recurrence rate of PV‐present and PV‐absent group (27.3% and 3.9% at 6 months, and 41.6% and 5.5% at 1 year, respectively; *p* < 0.001).

The cumulative recurrence rates after APC in the PV‐present group were significantly higher than those in the PV‐absent group (*p* < 0.001) (Figure 1). Furthermore, in the PV‐present and PV‐absent groups, the cumulative recurrence rates after APC were 27.3% and 3.9% at 6 months, and 41.6% and 5.5% at 1 year, respectively.

## 4 Discussion

Risk stratification and surveillance endoscopy at appropriate intervals are necessary to identify post‐treatment EV recurrence. Our study demonstrated that PVs detected using EUS 1 month after EVL combined with APC independently predicted EV recurrence. Furthermore, patients with PVs had a high cumulative recurrence rate of 27.3% at 6 months post‐treatment. Because this report appears to be the first to focus on the importance of EUS findings after EVL combined with APC, these findings provide important information to identify high‐risk populations for EV recurrence and determine the optimal follow‐up interval for these patients.

The first crucial result of our study is the usefulness of EUS findings, especially PVs, in stratifying the risk of EV recurrence after EVL combined with APC. Previous research targeting patients who underwent sclerotherapy or various treatments has shown that EV recurrence is significantly associated with the presence of PV and large peri‐Vs on EUS post‐treatment [4, 9]. However, few studies have focused on patients who underwent EVL‐based treatment, which is recommended in the guidelines as a prophylactic treatment for EV [7, 8, 14]. Notably, this is the first report on EVL combined with APC. Clarifying the relevance of EUS findings in patients treated with EVL combined with APC is essential because their risk factors, recurrence rate, and time to recurrence may differ from those associated with other therapies such as sclerotherapy [2, 15, 16]. Our study employed unified protocols for EVL and APC with a large cohort, making our findings clinically relevant, which are also consistent with those of previous research on the association between PVs and EV recurrence. Conversely, here, peri‐V was not a significant risk factor. Anatomically, EV recurrence after treatment results from peri‐V and para‐V blood flow entering the wall via the PV [17]; accordingly, the presence of the PV constitutes the sole EUS‐identified risk factor for EV recurrence. Our findings strongly indicate that conducting EUS and identifying PVs is a straightforward and promising approach for identifying patients at a high risk of EV recurrence following EVL combined with APC.

The second important result is the marked difference in cumulative recurrence rates between patients with and without PV. Generally, the Baveno VII consensus suggests the criteria for screening endoscopy based on liver stiffness and platelet counts [14]. Nonetheless, these criteria do not explain the risk factors for recurrence post‐treatment, and the optimal interval for surveillance endoscopy has not been specified. Although the ASGE and ESGE recommend surveillance EGD after EV eradication every 6–12 months and every 3–6 months, respectively [7, 8], evidence supporting this statement remains scarce. Notably, no previous studies, including those on EUS findings of esophageal collateral vessels, have compared the duration of recurrence based on the presence or absence of risk factors. Our analysis revealed an increased cumulative recurrence rate in patients with PVs (27.3% and 41.6% within 6 months and 1 year, respectively). Because more than 25% of patients with PVs experience recurrence within 6 months of treatment, we recommend performing EUS after EVL combined with APC and surveillance endoscopy every 6 months in the first year, especially for patients with PVs, to facilitate the early detection and treatment of EV recurrence and prevent rupture. Conversely, a 12‐month interval is acceptable for those without PVs as they exhibit a relatively low 12‐month recurrence rate of 5.5%. Ideally, all patients should be followed up with EUS after EVL combined with APC. Nevertheless, not all medical institutions possess the EUS equipment or well‐trained endoscopists, both crucial for performing EUS and detecting PVs. Given its versatility and minimal invasiveness, CT could become a widely available tool for assessing PV if dynamic CT proves capable of detecting them. However, because PV have an extremely small diameter and short length, no reports have demonstrated that CT can identify PV. Therefore, performing surveillance endoscopy every 6 months for all patients in the first year after EV treatment is a reasonable solution in daily practice.

Regarding additional pre‐recurrence treatments for patients with PV confirmed by EUS, no strategies specifically targeting PV have been reported. APC involves thermal coagulation limited to the mucosa or superficial submucosa, leading to mucosal fibrosis and suppression of capillary proliferation and PV invasion. Because APC does not occlude PV or alter hemodynamics, shorter surveillance endoscopy intervals in patients with PVs than in those without may be a reasonable approach, rather than adding intensive APC in these patients. Regarding the APC strategy, previous reports have described variations in session number and output levels, with recurrence rates ranging from 0% to 26% [2, 5, 6, 18]. Although direct comparison is difficult, we observed no marked difference in recurrence rates between our results and those reported previously. These findings indicate that consistent, thorough mucosal coagulation across the entire treatment area is essential for reproducible outcomes, regardless of treatment session number, device type or mode, or practitioner experience level. Additionally, removal of the coagulated mucosa using an endoscope equipped with a tip hood, along with additional coagulation of remaining non‐coagulated areas, may help achieve this goal.

Our study had some limitations. First, a single center in Japan can limit the generalizability of the results to other regions. Second, we lacked data on liver stiffness measurement and hepatic venous pressure gradient, both of which are important for stratifying the risk of EV recurrence. In our study, patients with EV recurrence had a significantly lower platelet count. Because platelet counts reflect liver stiffness, these patients likely had elevated liver stiffness and hepatic venous pressure gradients, which may have interacted with the remaining perforating veins. Additional limitations included the absence of vascular diameter measurement in EUS, EUS examination performed only after APC, and non‐uniform application of non‐selective beta‐blockers. Despite these limitations, the study has notable strengths, including being the first cohort of EVL combined with APC, a uniform protocol for treatment, and an analysis considering mortality as a competing risk, all of which are crucial for providing robust evidence to guide daily practice.

In conclusion, our study demonstrated that the presence of PV on EUS independently predicts EV recurrence in patients after EVL combined with APC. Because patients with PVs exhibit a high recurrence rate within 6 months, comprehensive surveillance endoscopy at 6‐month intervals is recommended in the first year post‐treatment. Future research is warranted to validate our findings in the context of EVL combined with APC and following stratification by EUS.

## Author Contributions

Yukari Tezuka: writing – original draft preparation (lead), formal analysis (lead). Jun Takada: writing – review and editing (lead), conceptualization (lead). Takao Miwa: formal analysis (equal). Kiichi Otani: writing – review and editing (supporting). Naoya Masuda: writing – review and editing (supporting). Hiroki Taniguchi: writing – review and editing (supporting); Kentaro Kojima: writing – review and editing (supporting). Sachiyo Onishi: writing – review and editing (supporting). Masaya Kubota: writing – review and editing (supporting). Takashi Ibuka: writing – review and editing (supporting). Masahito Shimizu: supervision (lead). All authors have approved the submitted version of the manuscript and agreed to be accountable for any part of this work.

## Funding

The authors have nothing to report.

## Ethics Statement

This study was approved by the Institutional Ethics Committee of the Gifu University Hospital (approval number: 2023‐242; approval date: January 17, 2024). The study was conducted in accordance with the principles of the Declaration of Helsinki. Given the retrospective nature of the study, informed consent was obtained from all the participants using an opt‐out method.

## Conflicts of Interest

The authors declare no conflicts of interest.

## Supporting information


**Figure S1:** Strategy of endoscopic procedure. Abbreviations: EVL, endoscopic variceal ligation; APC, argon plasma coagulation; EGD, esophagogastroduodenoscopy; EUS, endoscopic ultrasonography.

**Figure S2A:** Findings of endoscopic ultrasonography with miniature ultrasonic probe (perforating vein).

**Figure S2B:** Findings of endoscopic ultrasonography with miniature ultrasonic probe (peri‐esophageal vein and para‐esophageal vein).

**Figure S3:** Flowchart of study protocol. Abbreviations: EV, esophageal varices; EVL, endoscopic variceal ligation; APC, argon plasma coagulation; EUS, endoscopic ultrasonography.

**Table S1:** Baseline characteristics of patients who underwent variceal treatment, divided by treatment history.

**Table S2:** Multivariable model for predicting varices recurrence in treatment‐naive patients.

## Data Availability

Due to the nature of this research, participants of this study did not agree for their data to be shared publicly, so supporting data are not available.
