# Supplementary figures and images for: Perforating Veins Detected by Endoscopic Ultrasonography Are Useful in Predicting the Recurrence of Esophageal Varices After Endoscopic Variceal Ligation Combined With Argon Plasma Coagulation
Source: Dig Endosc. 2026 Mar 9;38(3):e70132. doi: 10.1111/den.70132 (PMC12972645; doi:10.1111/den.70132)

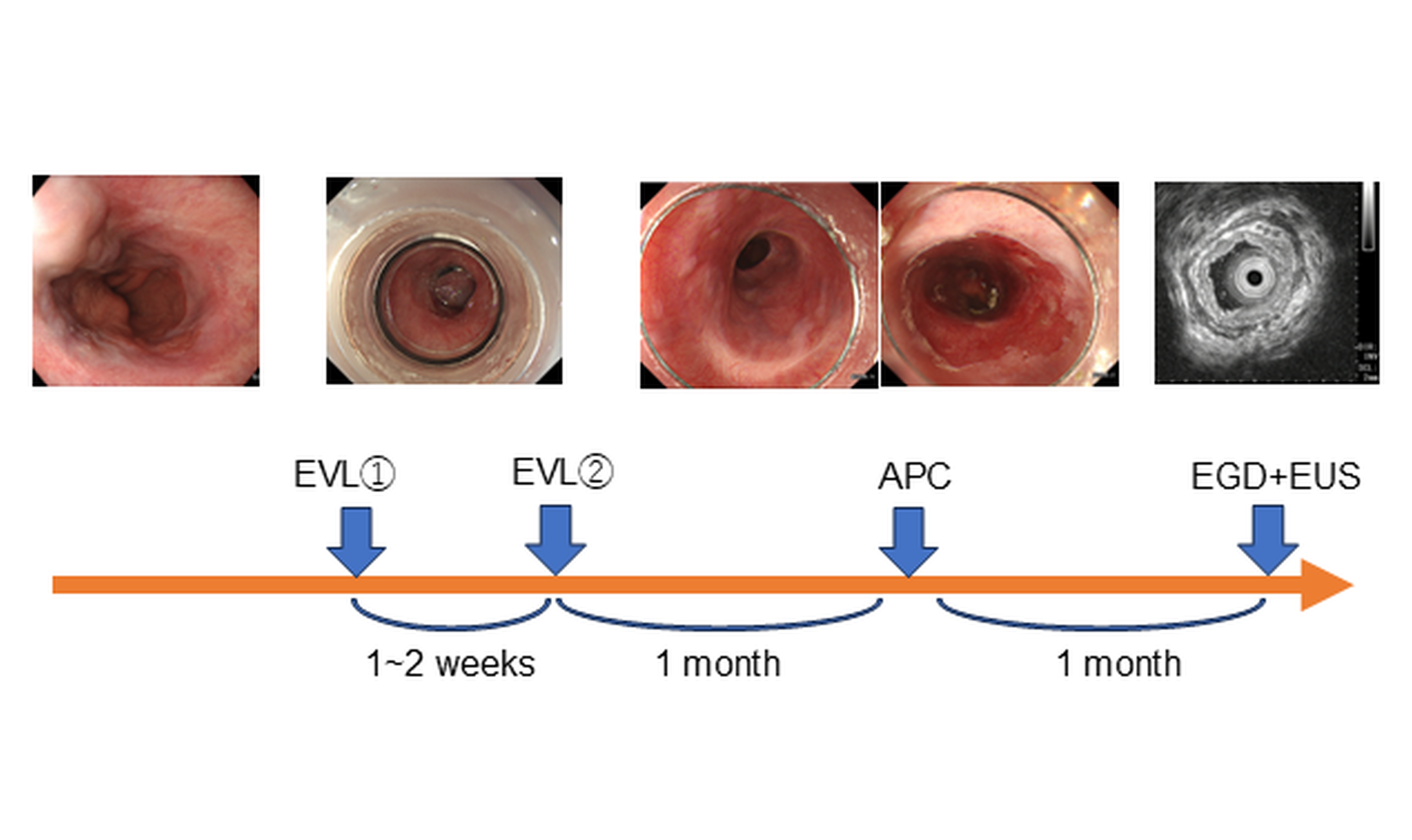

Supplement: Supplementary file 1 — Figure S1: Strategy of endoscopic procedure. Abbreviations: EVL, endoscopic variceal ligation; APC, argon plasma coagulation; EGD, esophagogastroduodenoscopy; EUS, endoscopic ultrasonography. [file DEN-38-0-s003.tif]

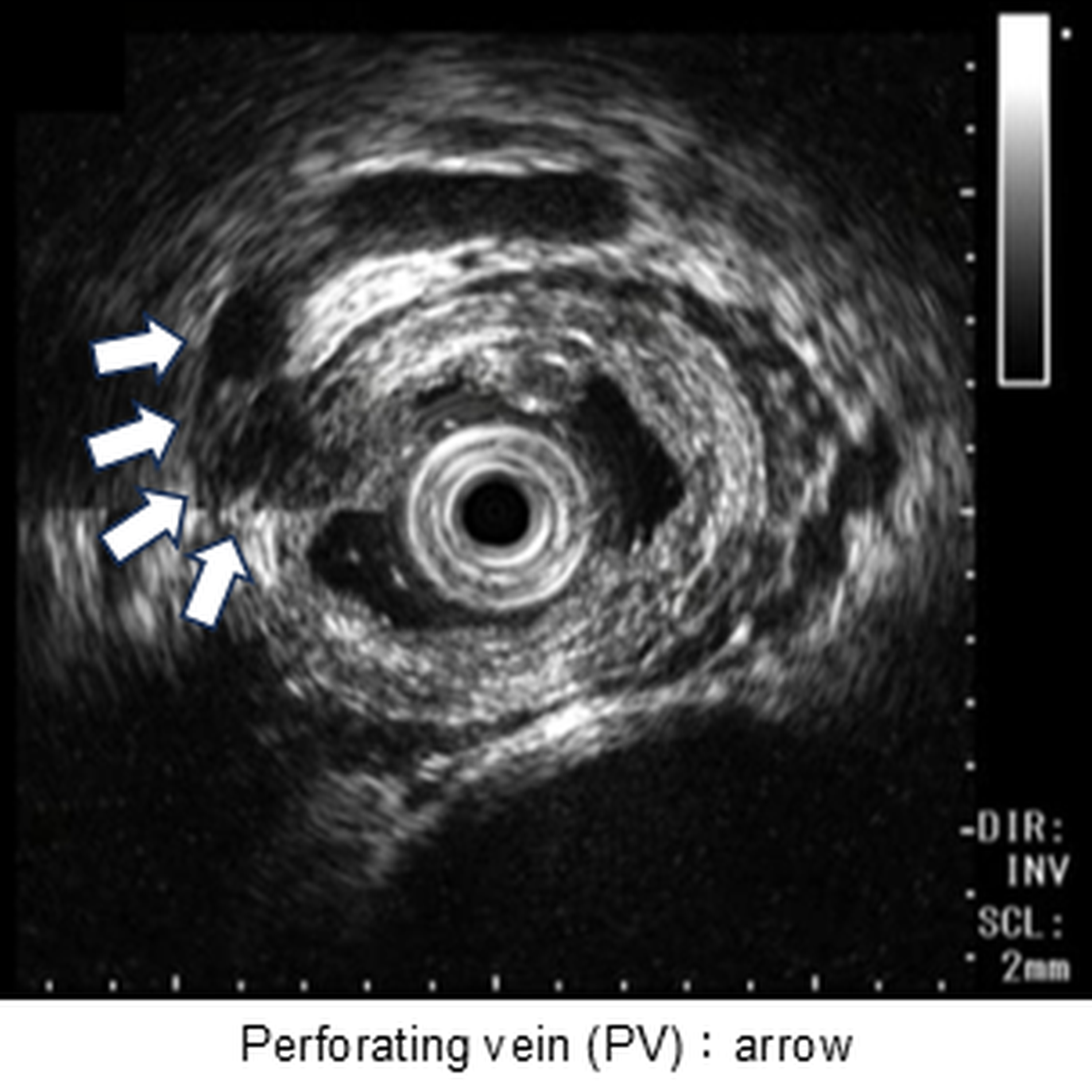

Supplement: Supplementary file 2 — Figure S2A: Findings of endoscopic ultrasonography with miniature ultrasonic probe (perforating vein). [file DEN-38-0-s005.tif]

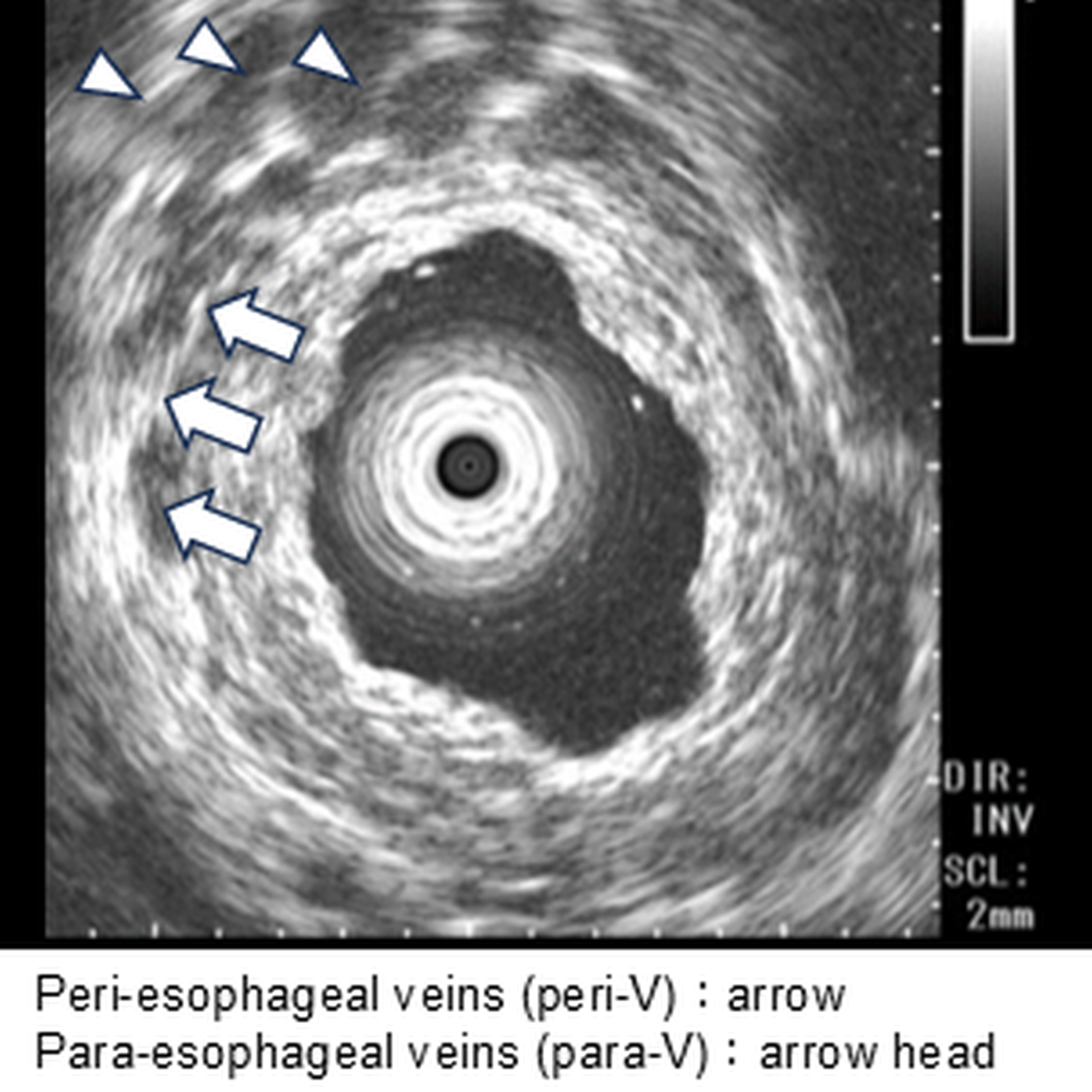

Supplement: Supplementary file 3 — Figure S2B: Findings of endoscopic ultrasonography with miniature ultrasonic probe (peri‐esophageal vein and para‐esophageal vein). [file DEN-38-0-s001.tif]

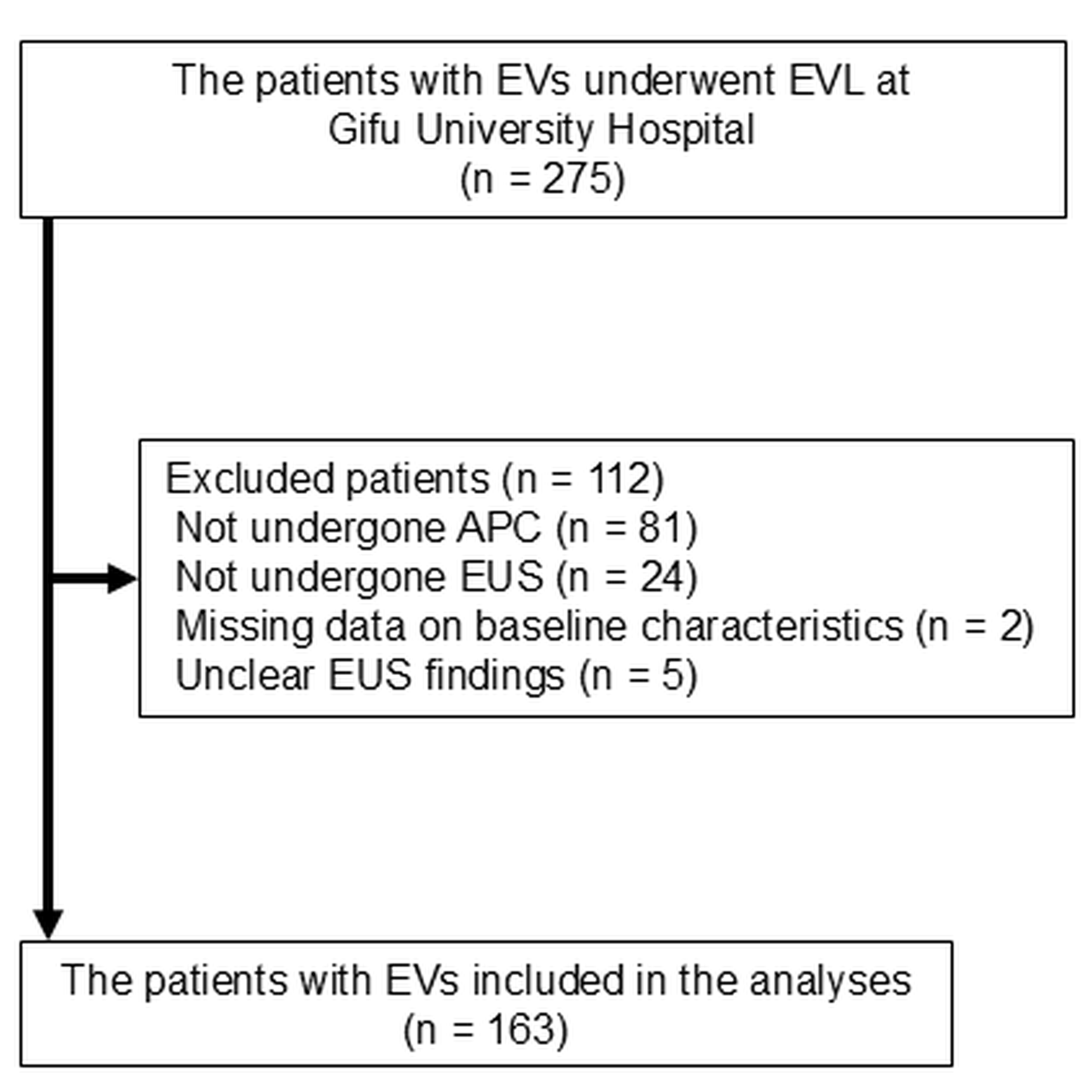

Supplement: Supplementary file 4 — Figure S3: Flowchart of study protocol. Abbreviations: EV, esophageal varices; EVL, endoscopic variceal ligation; APC, argon plasma coagulation; EUS, endoscopic ultrasonography. [file DEN-38-0-s006.tif]
