# Supplementary material for: Perforating Veins Detected by Endoscopic Ultrasonography Are Useful in Predicting the Recurrence of Esophageal Varices After Endoscopic Variceal Ligation Combined With Argon Plasma Coagulation
Source: Dig Endosc. 2026 Mar 9;38(3):e70132. doi: 10.1111/den.70132 (PMC12972645; doi:10.1111/den.70132)
Supplement: Supplementary file 5 — Table S1: Baseline characteristics of patients who underwent variceal treatment, divided by treatment history. [file DEN-38-0-s004.docx]

Table S1. Baseline characteristics of patients who underwent variceal treatment, divided by treatment history

| Characteristic | All patients | Treatment naive | Treatment experienced | *P-*value* |
| --- | --- | --- | --- | --- |
|  | (n = 163) | (n = 125) | (n = 38) |  |
| Age (years) | 67 (57–75) | 69.0 (58–76) | 61.0 (54–70) | 0.006 |
| Male, n (%) | 107 (66) | 80 (64) | 27 (71) | 0.559 |
| Body mass index (kg/m^2^) | 23.0 (20.6–25.7) | 23.0 (20.9–25.9) | 23.5 (20.3–25.1) | 0.953 |
| Etiology of cirrhosis, n (%) |  |  |  | 0.818 |
| Alcohol | 56 (34) | 41 (33) | 15 (40) |  |
| Viral | 43 (26) | 34 (27) | 9 (24) |  |
| Others | 64 (39) | 50 (40) | 14 (37) |  |
| Hepatocellular carcinoma, n (%) | 39 (24) | 31 (25) | 8 (21) | 0.828 |
| Portal vein thrombosis, n (%) | 16 (10) | 9 (7) | 7 (18) | 0.059 |
| Portosystemic shunts other than esophageal collateral veins, n (%) | 90 (55) | 67 (54) | 23 (61) | 0.577 |
| Paraumbilical veins | 50 (31) | 38 (31) | 12 (32) | 1.000 |
| Splenorenal shunts | 59 (36) | 43 (35) | 16 (42) | 0.444 |
| Gastrorenal shunt | 14 (9) | 10 (8) | 4 (11) | 0.741 |
| Mesenteric vein shunt | 6 (4) | 3 (2) | 3 (8) | 0.142 |
| Non-selective beta-blockers intake, n (%) | 2 (1) | 2 (2) | 0 (0) | 1.000 |
| Ascites (moderate or severe), n (%) | 47 (29) | 36 (29) | 11 (29) | 1.000 |
| Hepatic encephalopathy, n (%) | 2 (1) | 1 (1) | 1 (3) | 0.413 |
| Child–Pugh class (A/B/C), n | 102/53/8 | 76/42/7 | 26/11/1 | 0.688 |
| Child–Pugh score | 6 (5–7) | 6 (5–7) | 6 (5–7) | 0.981 |
| International normalized ratio | 1.09 (1.03– 1.19) | 1.10 (1.03– 1.19) | 1.08 (1.04– 1.18) | 0.911 |
| Platelet (10^9^/L) | 87 (60–120) | 92 (66–126) | 64 (51–94) | <0.001 |
| Creatinine (mg/dL) | 0.71 (0.59–0.85) | 0.74 (0.59–0.89) | 0.66 (0.58–0.75) | 0.057 |
| Albumin (g/dL) | 3.60 (3.20–3.90) | 3.60 (3.20–3.90) | 3.55 (3.30–4.07) | 0.361 |
| Bilirubin (mg/dL) | 1.30 (0.90–1.70) | 1.30 (0.90–1.70) | 1.30 (1.00–1.55) | 0.858 |
| EVs recurrence after treatment, n (%) | 37 (23) | 21 (17) | 16 (42) | 0.002 |
| Varices before treatment |  |  |  |  |
| Location (Li/Lm/Ls), n | 31/103/29 | 18/83/24 | 13/20/5 | 0.035 |
| Form (F1/F2/F3), n | 19/114/30 | 7/91/27 | 12/23/3 | <0.001 |
| Red color sign (0/1/2/3), n | 36/92/25/10 | 32/64/20/9 | 4/28/5/1 | 0.088 |
| Color (Cb/Cw), n | 150/13 | 118/7 | 32/6 | 0.079 |
| Rupture, n (%) | 16 (10) | 16 (13) | 0 (0) | 0.024 |
| Total number of EVL | 17 (14–24) | 19 (16–24) | 14 (9–17) | 0.005 |
| Total session of EVL | 2 (2–2) | 2 (2–2) | 2 (1–2) | 0.842 |
| EUS findings |  |  |  |  |
| Para-esophageal veins, n (%) | 123 (76) | 91 (73) | 32 (84) | 0.198 |
| Peri-esophageal veins, n (%) | 107 (66) | 81 (65) | 26 (68) | 0.846 |
| Perforating vein, n (%) | 22 (14) | 14 (11) | 8 (21) | 0.172 |

Values are presented as number (percentage) or median (interquartile range).

*Groups were compared using Fisher’s exact test or the Mann-Whitney *U* test.

Abbreviations: EV, esophageal varices; EUS, endoscopic ultrasound; EVL, endoscopic variceal ligation
