# Supplementary material for: Perforating Veins Detected by Endoscopic Ultrasonography Are Useful in Predicting the Recurrence of Esophageal Varices After Endoscopic Variceal Ligation Combined With Argon Plasma Coagulation
Source: Dig Endosc. 2026 Mar 9;38(3):e70132. doi: 10.1111/den.70132 (PMC12972645; doi:10.1111/den.70132)
Supplement: Supplementary file 6 — Table S2: Multivariable model for predicting varices recurrence in treatment‐naive patients. [file DEN-38-0-s002.docx]

Table S2. Multivariable model for predicting varices recurrence in treatment‑naive patients

| Characteristic | SHR (95% CI) | *P*-value^*^ |
| --- | --- | --- |
| Age (years) | 0.99 (0.97–1.02) | 0.610 |
| Male | 1.08 (0.41–2.89) | 0.870 |
| Child–Pugh score | 1.04 (0.83–1.30) | 0.740 |
| Platelet (10^9^/L) | 0.93 (0.84–1.02) | 0.120 |
| Peri-esophageal veins | 1.18 (0.45–3.13) | 0.730 |
| Perforating vein | 4.65 (1.94–11.16) | <0.001 |

* Analysis was performed using the fine-gray competing risk regression model.

Abbreviations: CI, confidence interval; SHR, sub-distribution hazard ratio
